# Patient Safety Climate: Variation in Perceptions by Infection Preventionists and Quality Directors

**DOI:** 10.1155/2011/357121

**Published:** 2011-08-04

**Authors:** Shanelle Nelson, Patricia W. Stone, Sarah Jordan, Monika Pogorzelska, Helen Halpin, Megan Vanneman, Elaine Larson

**Affiliations:** ^1^Center for Health Policy, Columbia University School of Nursing, New York, NY 10032, USA; ^2^Center for Health and Public Policy Studies, University of California Berkeley School of Public Health, 50 University Hall no. 7360, Berkeley, CA 94720, USA

## Abstract

*Background*. Healthcare-associated infections (HAIs) are an important patient safety issue, and safety climate is an important organizational factor. This study explores perceptions of infection preventionists (IPs) and quality directors (QDs) regarding two safety microclimates, Senior Management Engagement (SME) and Leadership on Patient Safety (LOPS), across California hospitals. *Methods*. This was an analysis of two cross-sectional surveys. We conducted Wilcoxon signed-rank test, univariate analyses, and a multivariate ordinary least square regression. *Results*. There were 322 eligible hospitals; 149 hospitals (46.3%) responded to both surveys. The IP response rate was 59%, and the QD response rate was 79.5%. We found IPs perceived SME more positively than did QDs (21.4 vs. 20.4, *P* < 0.01). No setting characteristics predicted variation in perceptions. Presence of an independent budget predicted more positive perceptions of microclimates across personnel types (*P* < 0.01). *Conclusions*. Differences in perceptions continue to exist between essential leaders in acute health care settings which could have critical effects on outcomes such as HAIs. Having an independent budget for the infection prevention and control department may enhance the overall safety climate and in turn patient care.

## 1. Introduction

It has been a decade since the spotlight on preventable medical errors first brought much needed attention to the “culture of safety” in health care organizations [1]. A “culture of safety” has been defined as the shared values and patterns of behavior that determine the degree to which all organizational members direct their attention and action towards minimizing patient harm [2]. Many healthcare institutions have adopted a “culture of safety” philosophy as an integral part of their delivery process or service [3]. The perceptions and attitudes of personnel working in an organization about the safety culture are often termed “safety climate,” and can provide an important indication of the level of its safety culture [4]. Patient safety climate is a multidimensional phenomenon, and important microclimates have been identified such as senior management engagement and leadership [5, 6].

Previously, researchers have found that there are differences in attitudes and perceptions of safety climate among employees in various work areas and members of different disciplines. For example, Singer and colleagues reported that Emergency Department (ED) personnel, particularly ED nurses, perceived substantially lower levels of safety climate than workers in other areas of the hospital [7]. This group also found that the higher up the person is in the organizational hierarchy, the more safe they perceive the climate to be and that these perceptual differences are damaging to improving patient safety [7]. 

Healthcare-associated infections (HAI) are an important patient safety issue. In the past 20 years, the overall incidence of HAI has increased by 36%, and the substantial human suffering and financial burden of these infections is staggering [1]. Annually, in the United States, approximately 2 million patients develop an HAI, and nearly 90,000 of these patients are estimated to die; this ranks HAI as the fifth leading cause of death in acute care hospitals [8]. The Centers for Disease Control and Prevention has recently estimated the annual hospital costs of HAI in the U.S. to be between 25.0 to 31.5 billion dollars per year [9]. Because of the staggering costs and associated morbidity and mortality of this largely preventable problem, there has been a major focus on the reduction of HAI internationally [10]. The prevention and control of infections in a hospital is a quality-improvement activity that centers on improving the care of patients and protecting the health of staff [11, 12]; therefore, it would seem essential for there to be strong leadership with common goals in tackling these efforts. 

With a shift toward prevention and surveillance of HAI occurrences, the roles and responsibilities of those working in the field of infection prevention and control are expanding [13], and infection control professionals are now referred to as “infection preventionists” (IPs) [14]. IPs are responsible for directing interventions that protect patients from HAI and working with clinicians, staff, and administrators to improve patient- and systems-level outcomes to reduce HAI and other related adverse events [14]. IPs vary in educational background, but most have a nursing background [15, 16]. Often, quality directors (QDs) are also involved in the prevention of HAI. The QD role is often more diverse, but the individual should be familiar with the activities of the IPs as well as report to or be part of the senior leadership in the hospital. In terms of supporting the hospital infection program, the quality assurance department may be less responsible for continual monitoring and surveillance of HAI and more involved in outbreak investigation and exploring root causes [11, 12].

The present study explored whether patient safety climate varied between two different but essential roles in the prevention of infection and across different hospitals: infection preventionists (IPs) and quality directors (QDs). The aims of this study were to (1) compare the perceptions of two aspects of patient safety climate between IPs and QDs in the same hospital, (2) identify setting and role characteristics associated with differences in perceptions of patient safety climates, and (3) identify setting characteristics that predict more positive perceptions of patient safety climates. Given the differences in responsibility and fit in the hierarchy of hospitals, we hypothesize that IPs would perceive a lower climate of patient safety compared to QDs.

## 2. Methods

### 2.1. Design

This study was an analysis of two cross-sectional surveys conducted simultaneously in the Fall of 2008. Both surveys were conducted to obtain preliminary baseline data on different aspects related to the prevention of HAI with the ultimate goal of evaluating the California Healthcare-Associated Infection Prevention Initiative (CHAIPI) [17]. This statewide initiative was designed to reduce HAI using technology and educational sessions for IP personnel [17]. Additionally, hospitals formed an educational collaboration and shared knowledge, successes, and barriers to reducing HAI through webinars and meetings [17]. One survey was conducted by Columbia University in partnership with the Association for Professionals in Infection Control and Epidemiology (APIC) and targeted IPs; the other survey was conducted by researchers at The University of California Berkeley, Center for Health and Public Policy Studies, who surveyed the QDs.

### 2.2. Sample Eligibility, Recruitment, and Survey Processes

The IP and QD of all general acute care California hospitals were eligible to participate in their respective surveys. A list of all hospitals licensed to operate in California was obtained from the California Office of Statewide Health Planning and Development. Specialty hospitals and hospitals in which patients had a mean length of stay of ≥30 days were excluded. For the IP survey, the eligible hospital list was cross-referenced with APIC memberships to identify the IP at each of the hospitals. The IP survey was web based, and respondents were recruited using a modified Dillman technique [18], which included e-mail and mail invitations and reminders, as well as announcements included in regularly scheduled APIC communication materials (e.g., newsletters to members). For each QD, a structured, computer-assisted telephone interview was scheduled in advance and conducted with the QD of each hospital. When needed, multiple attempts were made to schedule the interview with each QD.

### 2.3. Variables

Both surveys included the same two measures of patient safety climate, which were adapted from the Patient Safety Climate in Healthcare Organizations (PSCHO) [6]. The first measure was the Senior Management Engagement scale, which measured the understanding of current safety issues in their facility, taking supportive action when necessary, and appreciating that frontline care providers are often best qualified to solve patient safety issues (e.g., senior management supports a climate that promotes patient safety) [6]. The second measure was the Leadership on Patient Safety scale, defined as the senior executives' ability to articulate values consistent with patient safety and reducing HAI (e.g., the senior executives clearly articulate the hospital values relevant to patient safety and HAI) [6]. Both the Senior Management Engagement and Leadership on Patient Safety scales included 5 items and used a 5-point Likert scale (1-Strongly Disagree to 5-Strongly Agree). The composite scores for each scale were calculated by summing the responses for each item with higher scores indicating a more positive perception of the safety climate. Cronbach's Alphas for each scale were: Senior Management Engagement *α* = .896 for the IPs and *α* = .902 for the QDs; Leadership on Patient Safety *α* = .931 for the IPs and *α* = .863 for the QDs.

Other data obtained include hospital characteristics (i.e., medical school affiliation, number of beds, and location of hospital—urban setting, suburban setting, rural setting), and infection prevention, and control department characteristics (i.e., independent budget of the department, presence of a physician hospital epidemiologist, use of an electronic surveillance system for infection detection, to whom the IP director reports to, IP respondent role, and number of IP fulltime equivalent (FTE) staff per 100 beds).

### 2.4. Statistical Analyses

All statistical analyses were performed using SPSS version 17. Descriptive statistics (i.e., frequencies, means, and standard deviations) were computed on all variables. Histograms were generated for each of the continuous variables to examine normality. Single missing items per case on the Senior Management Engagement or the Leadership on Patient Safety scales were imputed. Imputation consisted of taking the average score of the other four items. 

For the first aim, we examined differences in IP and QD perceptions on both the items and composite patient safety microclimate scales using the Wilcoxon signed-rank test. To meet the second aim, univariate analyses using *t*-tests were conducted. For the third aim, we developed a multivariate ordinary least square regression to examine the association between setting characteristics and perceptions of both patient safety scales for both personnel types. The final models included the hospital and infection prevention and control department characteristics that were associated with the scores in univariate analyses with *P* ≤ 0.1.

## 3. Results

### 3.1. Sample Recruited

There were 322 eligible hospitals; 149 hospitals (46.3%) responded to both surveys. Seventeen hospitals with insufficient data were excluded. Additionally, hospitals were removed from specific analyses if there were two or more missing responses and imputation was not possible. Therefore, the final sample size for the Senior Management Engagement scale was 129 and 132 for the Leadership on Patient Safety scale. 

Descriptive statistics for hospital characteristics are provided in Table 1. Most hospitals were located in an urban setting (42%), followed by suburban (33%) or rural settings (34%). The average bed size of participating facilities was 241 (SD ± 161, range: 25–952). 

In both the Senior Management Engagement and Leadership on Patient Safety scales, four of five items on each scale were significantly different (Table 2). IPs perceived the senior management items more positively than the QDs (difference in mean scores ranged from 0.2 to 0.3, all *P* values ≤ 0.05). Additionally, IPs' perceptions were more positive than the QDs on the Senior Management Engagement composite score (21.4 versus 20.4, *P* < 0.01). The QDs' perceptions were more positive than the IPs' on the Leadership on Patient Safety items (difference in mean scores ranged 0.2 to 0.3, all *P* values ≤ 0.05); however, there was no difference on the composite score. 

None of the hospital and infection prevention and control department setting characteristics were significant predictors of the differences between the IPs and QDs perceptions of either of the safety microclimates. However, the IP respondents' that identified themselves as Directors of the infection prevention and control department perceived more positive Senior Management Engagement compared to IP NonDirectors (mean 21.8, SD = 3.8 versus mean 20.5, SD = 4.3, *P* = 0.042). No role characteristics were associated with differences in perceptions on the Leadership on Patient Safety scale.

The results of the univariate analyses are displayed in Table 3. An independent budget for the infection prevention and control department was a significant predictor of more positive perceptions of patient safety in all 4 regressions. The presence of a hospital epidemiologist was also a significant predictor among QDs of more positive perceptions on the Leadership on Patient Safety scale. The number of hospital beds predicted more positive perceptions among QDs on both scales. When entered into multivariate linear regression (Table 4), having an independent budget remained the only statistically significant predictor of a high score for both IPs and QDs on both scales in each of the four regression models.

## 4. Discussion

This study examined the perceptions of two important patient safety microclimates from two different hospital personnel roles engaged in patient safety involving the prevention of infections. We found that IPs and QDs in the same hospital varied in their perceptions across the two patient safety climate scales. Our hypothesis that IPs would perceive a lower climate of patient safety compared to QDs was supported in only one of the microclimates. Generally, IPs had more positive perceptions of Senior Management Engagement and the QDs had more positive perceptions of Leadership on Patient Safety. We also found that having an independent budget for the infection prevention program was the only significant predictor of these microclimates. 

One reason for the differences in perceptions by personnel may be the measures of the microclimates themselves. For example, the items in the Leadership on Patient Safety scale were more tailored to HAIs. While IPs are generally involved with overall quality and patient safety, their primary role and interest is in prevention and control of infections. This could explain why the IPs may have more negative perceptions about the way hospital executives handle improvements in infection prevention and control. This may also be due to the Senior Management Scale being less tailored to infection prevention. 

Although there were no setting characteristics that were significant predictors of differences between IPs and QDs, IPs who were Directors of their departments perceived the Senior Management Engagement more positively than IPs who were not Directors. In a study of personnel in 92 hospitals, Singer and colleagues [19] found differences in perceptions of safety climate by both role (i.e., senior management, supervisor, and front line worker) and by discipline (i.e., physician, nurse, other clinician and nonclinician). Similar to our findings, these researchers found that senior managers perceived fewer problems with Senior Management Engagement than front line workers. 

However, another group of researchers found that leadership played a key role in infection prevention and that the most important leaders were not the senior executives traditionally associated with the term “leader” [20]. They found several examples of hospital epidemiologists, nurses, quality managers, and infection preventionists who played vital roles in their hospital's patient safety activities [20]. Finding ways to empower all IPs to be “leaders” in patient safety is likely to be an important factor in reducing infections. 

Another key finding of our study is that budget was an important predictor of more positive perceptions of patient safety climates. Having an independent budget for the infection prevention and control department may allow for more autonomy and development of infrastructure to promote patient safety. According to a policy brief by Pronovost et al., efforts are being made at Johns Hopkins Hospital to improve the safety culture by investing resources to monitor the rate-based measures of quality and safety, nearly all of which are required by the Centers for Medicaid and Medicare Services (CMS), The Joint Commission, or insurers [21]. These authors noted that fulfilling a commitment to safe and high-quality care is not possible without significant investment in patient safety infrastructure. Based on a study by Fukuda et al., implementing hospital-wide safety practices requires considerable financial investment [22]. Results from their study confirmed that hospitals with greater financial and organizational resources are more capable of promoting the activities required for patient safety and infection control [22].

As with any research, this study has both strengths and limitations. Using well-developed psychometrically tested measures of safety climate is a strength. In terms of internal validity, the data were obtained using two different methods for each personnel type. It is known that the survey mode can make a difference in responses, and in several studies researchers have found that telephone respondents answer questions more positively than do mail respondents [18, 23, 24]. Additionally, in a study by Christian et al., telephone respondents gave significantly more positive answers than did web respondents for various kinds of scale questions [25]. However, the fact that the QDs, who completed the survey via computer-assisted telephone, scored higher on one scale and not the other provides some evidence that it was unlikely that there was bias due to survey method. 

Another limitation is the study design. This was a cross sectional study, and only associations may be examined, not causation. Both surveys were conducted in the Fall of 2008. At the time the surveys were conducted, California hospitals were preparing for mandatory reporting of HAI to the state's health department, and the CMS policy on lack of reimbursement for hospital-acquired conditions (including many types of infections) was just being implemented [26]. Therefore, these results may not be generalizable to other hospitals outside California. Our response rate is typical of multisite surveys of hospital personnel, which often have response rates in the range from 40 to 50 percent [27]. Lastly, in this analysis only patient safety climate was measured. Further analyses are needed to examine how these differences in climate impact the processes of care and patient outcomes.

## 5. Conclusions

 Although there have been many efforts to curb the increase in HAI, it is clear that this preventable issue is slow to improve. Leaders play a pivotal role in hospital initiatives to improve quality. This study represents an advance over previous studies on the relationship between safety climate and personnel perceptions by examining those leaders who are essential to the prevention of HAIs in acute health care settings. It also provides a solid basis for subsequent research on decreasing the gap in perceptions between these two personnel types. Given the finding that there are differences in perceptions among essential leaders, this discord could be an inhibition toward achieving the goal of decreased HAIs. It is essential for those personnel in leadership to work collaboratively in order to not only enhance health care environments but also make it safer for patients. 

This paper also highlights the importance of independently supporting the infection prevention and control department in order to optimize the safety climate. This is important when discussing ideas and ways to curtail the overall HAI issue.

## Figures and Tables

**Table 1:** Hospital and infection prevention and control department characteristics.

	*N* = 132	%
Medical school affiliation		
Yes	28	21
No	104	79
Location of hospital		
Urban setting	55	42
Suburb	43	33
Rural setting	34	26
Infection Prevention Program has independent budget		
Yes	70	53
No/missing	62	47
Presence of hospital epidemiologist		
Yes	68	52
No/missing	64	48
Use of an electronic surveillance System		
Yes	37	28
No	95	72
Infection Prevention Director reports to Quality Management Director/Vice President or Director/Vice President of Patient Safety		
Yes	27	20
No	105	80
Role in infection prevention department (IPs only)		
IP Director/coord.		
Yes	99	75
No/missing	33	25

	Mean (SD)	

Beds	241 (±161)	
Number of infection prevention professional staff per 100 beds	0.55 (±0.56)	

**Table 2:** Patient safety climates by personnel type.

Variable	IP-mean	IP-Std. Dev	QD-mean	QD-Std. Dev	Difference in mean scores	*P*-value
Senior Management Engagement (SME)						

Senior management supports a climate that promotes patient safety.	4.5	0.8	4.3	0.6	0.2	<0.01

Senior management has a clear picture of the risks associated with patient care.	4.3	1.0	4.1	0.7	0.2	0.05

Senior management has a good idea of the kinds of mistakes that actually occur in this hospital.	4.4	0.9	4.2	0.8	0.2	<0.01

Senior management considers patient safety when program changes are discussed.	4.3	0.9	4.0	0.8	0.3	<0.01

Good communication flow exists up and down the chain of command regarding patient safety issues.	3.9	1.1	3.8	0.8	0.1	**0.15**

Leadership on Patient Safety (LOPS)						

The senior executives clearly articulate the hospital's values relevant to patient safety and healthcare-associated infections.	4.0	1.1	4.2	0.8	0.2	0.03

The behavior of the senior executives is consistent with values relevant to patient safety and healthcare-associated infections.	3.9	1.1	4.2	0.7	0.3	0.01

The senior executives have demonstrated an ability to manage the changes (e.g., organizational, technological) needed to improve patient safety and reduce healthcare-associated infections.	3.7	1.2	3.9	0.7	0.2	0.02

The senior executives act on suggestions to improve patient safety and reduce healthcare-associated infections.	4.0	1.0	4.0	0.7	0	**0.95**

The hospital's senior executives generate confidence that efforts to improve patient safety and reduce healthcare-associated infections will succeed.	3.9	1.1	4.1	0.7	0.2	0.04

Composite scores						

Senior Management Engagement	21.4	3.9	20.4	2.9	1.1	<0.01
Leadership on Patient Safety	19.5	4.9	20.4	3.0	0.9	**0.13**

IP: infection preventionist; QD: quality director.

**Table 3:** Regression coefficients from univariate analyses of hospital and infection prevention and control department characteristics and SME/LOPS scores.

Variable	SME				LOPS			
	IP		QD		IP		QD	
	b (SE)	*P* value	b (SE)	*P* value	b (SE)	*P* value	b (SE)	*P* value
Medical school affiliation	−0.196 (.855)	0.819	0.516 (0.628)	0.412	0.508 (1.068)	0.635	0.640 (0.647)	0.32

Infection Prevention Program has independent budget	**2.11 (.671)**	**0.002**	**1.657 (0.495)**	**0.001**	**2.428 (0.852)**	**0.005**	**1.652 (0.512)**	**0.00**

Presence of hospital epidemiologist	0.456 (.695)	0.513	0.523 (0.513)	0.31	0.738 (0.877)	0.401	**1.066 (0.523)**	**0.04**

Use of an Electronic Surveillance System	0.520 (.774)	0.503	0.600 (0.571)	0.295	0.951 (0.959)	0.323	0.086 (0.591)	0.88

Infection Prevention Director reports to Quality Management Director/Vice President or Director/Vice President of Patient Safety	−0.241 (0.738)	0.744	−0.734 (0.545)	0.181	−1.034 (0.932)	0.269	−0.008 (0.566)	0.99

Beds	0.002 (.002)	0.447	**0.003 (0.002)**	**0.072**	0.002 (0.003)	0.495	**0.003 (0.002)**	**0.10**

Number of infection prevention professional staff per 100 beds	−0.672 (.664)	0.313	−0.355 (0.472)	0.453	−0.544 (0.826)	0.511	−0.541 (0.483)	0.27

IP: infection preventionist; QD: quality director; SME: Senior Management Engagement; LOPS: Leadership on Patient Safety.

**Table 4:** Multivariable regression models of hospital characteristics and SME/LOPS scores.

Variable	SME				LOPS			
	IP		QD		IP		QD	
	b (SE)	*P* value	b (SE)	*P* value	b (SE)	*P* value	b (SE)	*P* value
Infection Prevention Program has independent budget	**2.064 (0.680)**	**0.003**	**1.577 (0.496)**	**0.002**	**2.358 (0.863)**	**0.007**	**1.531 (0.509)**	**0.003**

Presence of Hospital Epidemiologists	0.185 (0.691)	0.789	0.239 (0.503)	0.636	0.417 (0.877)	0.636	0.814 (0.516)	0.118

Beds	0.001 (0.002)	0.631	0.002 (0.002)	0.136	0.001 (0.003)	0.684	0.002 (0.002)	0.24

IP: infection preventionist; QD: quality director; SME: senior management; LOPS: leadership on patient safety.
